# ManiNetCluster: a novel manifold learning approach to reveal the functional links between gene networks

**DOI:** 10.1186/s12864-019-6329-2

**Published:** 2019-12-30

**Authors:** Nam D. Nguyen, Ian K. Blaby, Daifeng Wang

**Affiliations:** 1 Deparment of Computer Science, Stony Brook University, Stony Brook, NY 11794 USA; 2 Biology Department, Brookhaven National Laboratory, Upton, NY 11973 USA; 3 US Department of Energy, Joint Genome Institute, Lawrence Berkeley National Laboratory, 1 Cyclotron Road, Berkeley, 4720 CA USA; 4 Department of Biostatistics and Medical Informatics, University of Wisconsin-Madison, Madison, 53726 WI USA; 5 Waisman Center, University of Wisconsin-Madison, Madison, 53705 WI USA

**Keywords:** Manifold learning, Manifold regularization, Clustering, Multiview learning, Functional genomics, Comparative network analysis, Comparative genomics, Biofuel

## Abstract

**Background:**

The coordination of genomic functions is a critical and complex process across biological systems such as phenotypes or states (e.g., time, disease, organism, environmental perturbation). Understanding how the complexity of genomic function relates to these states remains a challenge. To address this, we have developed a novel computational method, ManiNetCluster, which simultaneously aligns and clusters gene networks (e.g., co-expression) to systematically reveal the links of genomic function between different conditions. Specifically, ManiNetCluster employs manifold learning to uncover and match local and non-linear structures among networks, and identifies cross-network functional links.

**Results:**

We demonstrated that ManiNetCluster better aligns the orthologous genes from their developmental expression profiles across model organisms than state-of-the-art methods (*p*-value <2.2×10^−16^). This indicates the potential non-linear interactions of evolutionarily conserved genes across species in development. Furthermore, we applied ManiNetCluster to time series transcriptome data measured in the green alga *Chlamydomonas reinhardtii* to discover the genomic functions linking various metabolic processes between the light and dark periods of a diurnally cycling culture. We identified a number of genes putatively regulating processes across each lighting regime.

**Conclusions:**

ManiNetCluster provides a novel computational tool to uncover the genes linking various functions from different networks, providing new insight on how gene functions coordinate across different conditions. ManiNetCluster is publicly available as an R package at https://github.com/daifengwanglab/ManiNetCluster.

## Background

The molecular processing that links genotype and phenotype is complex and poorly characterized. Understanding these mechanisms is crucial to comprehend how proteins interact with each other in a coordinated fashion. Biologically-derived data has undergone a revolution in recent history thanks to the advent of high throughput sequencing technologies, resulting in a deluge of genome and genome-derived (e.g., transcriptome) datasets for various phenotypes. Extracting all significant phenomena from these data is fundamental to completely understand how dynamic functional genomics vary between systems (such as environment and disease-state). However, the integration and interpretation of systems-scale (i.e., ‘omics’) datasets for understanding how the interactions of genomic functions relate to different phenotypes, especially when comparatively analyzing multiple datasets, remains a challenge.

Whereas the genome and the encoded genes are near-static entities within an organism, the transcriptome and proteome are dynamic and state-dependent. The relative quantity of each mRNA and protein species, defining the transcriptome and proteome respectively, function together as networks to implement biological functions. Such networks provide powerful models allowing the analysis of biological datasets; e.g., gene co-expression networks, derived from transcriptomes, are frequently used to investigate the genotype-phenotype relationships and individual protein function predictions [1–5]. To discover the functional network components, clustering methods have been widely used to detect the network structures that imply functional groupings among genes (e.g., gene co-expression modules) [2]. Clustering could be seen as grouping together similar objects; therefore, the key factor to consider first is the distance metric. Previous studies have suggested that some specific distance metrics are only suitable for some certain algorithms and vice versa [6–9]; e.g., *k-*means algorithm works effectively with Euclidean distance in low dimensional space but not for high dimensional one such as gene expression datasets [6, 9]. More importantly, genes in the network highly likely interact with each other locally in a non-linear fashion [10]; many biological pathways involve the genes with short geodesic distances in gene co-expression networks [11]. However, a variety of state-of-art methods cluster genes based on the global network structures; e.g., scale-free topology by [2]. Thus, to model local non-linear gene relationships, non-linear metrics including geodesic distance on a manifold have been used to quantify the similarity between genes and find the non-linear structures of gene networks [12]. In practice, k-nearest neighbor graphs (kNNGraphs) are often used to approximate the manifold structure [12].

While network analysis is a useful tool to investigate the genotype-phenotype relationships and to derive the biological functional abstraction (e.g., gene modules), it is hard to understand the relationships between conditions, and, in particular between different experiments (e.g., organisms, environmental perturbations). Therefore, comparative network analyses have been developed to identify the common network motifs/structures preserved across conditions that may yield a high-level functional abstraction. A number of computational methods have been developed to aid biological network, and comparative network analysis [2, 5, 13]. However, these methods typically rely on external information and prior knowledge to link individual networks and find cross-network structures such as counting shared or orthologous genes between cross-species gene co-expression networks [14]. Consequently, they potentially miss the unknown functional links that can happen between different gene sets. For example, the genes that express at different stages during cell fate and differentiation can be co-regulated by common master regulators [15, 16]. Additionally, in many cases that the datasets for different conditions are generated independently, individual networks constructed from these datasets of individual potentially have the network structures that are driven by data biases rather than true biological functions. To address this, a comparative method to uniformly analyze cross-condition datasets is essential.

To help overcome some of these limitations, we have developed a manifold learning-based approach, ManiNetCluster, to simultaneously align and cluster gene networks for comparative network analysis. ManiNetCluster enables discovery of inter-network structures implying potential functional linkage across gene networks. This method addresses the challenges for discovering (1) non-linear manifold structures across gene expression datasets and (2) the functional relationships between different gene modules from different datasets. Manifold learning has been successfully used to find aligned, local and non-linear structures among non-biological networks; e.g., manifold alignment [17, 18] and warping [19]. Previous efforts have resulted in tools that combine manifold learning and gene expression analysis [20], or to bring together manifold learning and simultaneous clustering [21]. However, to our knowledge, ManiNetCluster is the first which integrates manifold learning, comparative analysis and simultaneous network clustering together to systematically reveal genomic function linkages across different gene expression datasets. ManiNetCluster is publicly available as an R package at https://github.com/daifengwanglab/ManiNetCluster with an online tutorial (Additional file 3: Tutorial).

ManiNetCluster is a network embedding method to solve the network alignment problem, which aims to find the structure similarities between different networks. Due to the NP-completeness of the sub-graph isomorphism problem, state-of-the-art network alignment methods often requires heuristic approaches, mapping nodes across networks to maximize a “topological” cost function, e.g., S^3^ (symmetric substructure score) measure of static edge conservation [22] and static graphlet-based measure of node conservation [22, 23], PageRank based cost function and Markovian alignment strategies [24–26]. Unlike these topological approaches, which is based on network structure, ManiNetCluster is a subspace learning approach, embedding the nodes across different networks into a common low dimensional representation such that the distances between mapped nodes as well as the "distortion" of each network structure are minimized. We have achieved this by implementing manifold alignment [17, 18] and manifold co-regularization [27]. Recent works [28, 29] which also employ node embedding methods are similarity-based representation, relying on a fixed reproducing kernel Hilbert space. In contrast, our method is a manifold-based representation [30] being able to capture and to transform any arbitrary shape of the inputs. Furthermore, the fusion of networks in a common latent manifold allows us to identify not only conserved structure but also functional links between networks, highlighting a novel type of structure.

## Methods

**Fig. 1:**
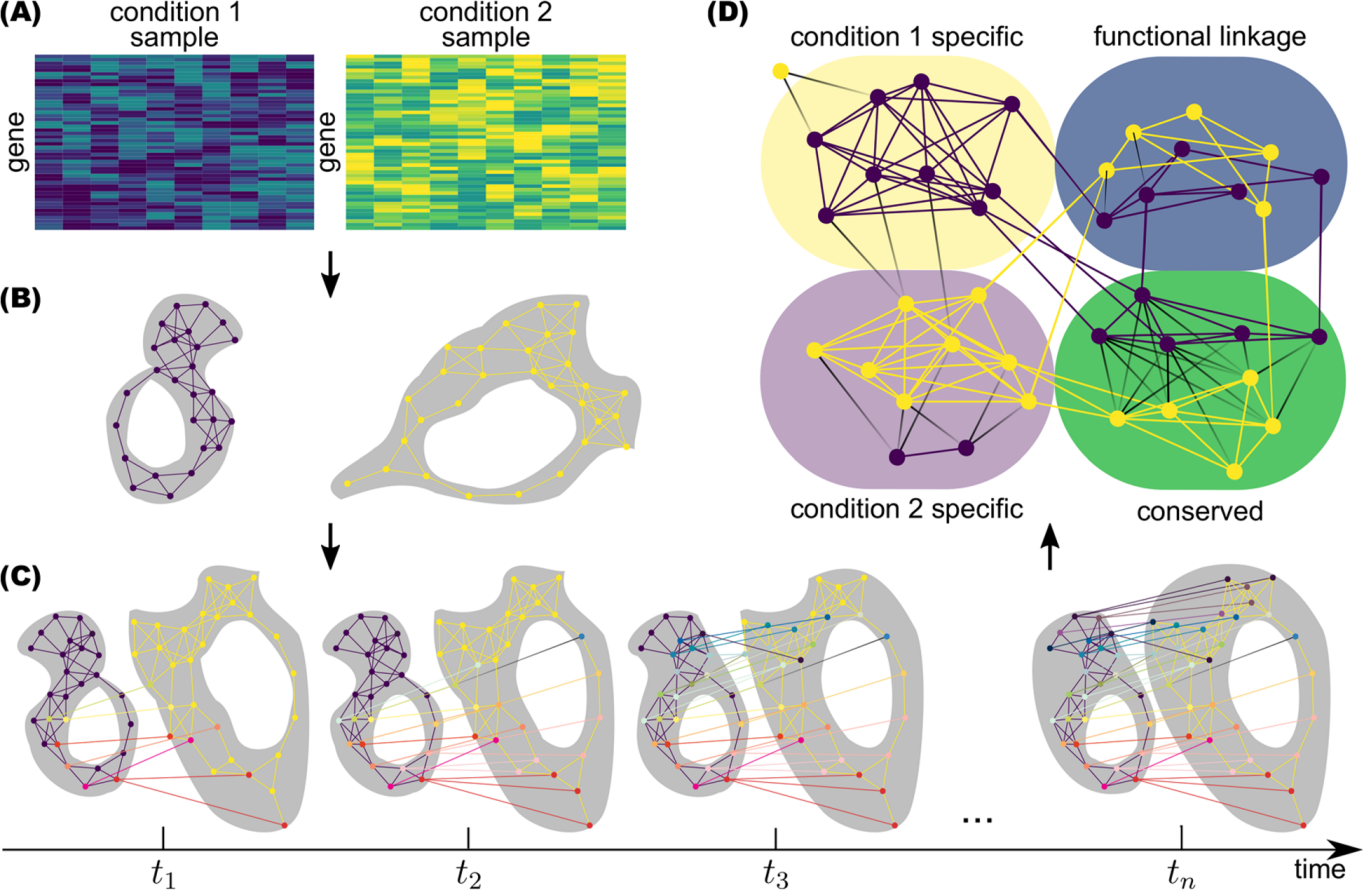
ManiNetCluster Workflow. **a** Inputs: The inputs of ManiNetCluster are two gene expression datasets collected from different phenotypes, states or conditions. **b** Manifold approximation via neighborhood networks: ManiNetCluster constructs gene co-expression network using kNNGraph for each condition, connecting genes with similar expression level. This step aims to approximate the manifolds of the datasets. **c** Manifold learning for network alignment: Using manifold alignment and manifold warping methods to identify a common manifold, ManiNetCluster aligns two gene networks across conditions. The outcome of this step is a multilayer network consisting of two types of links: the inter-links (between the two co-expression neighborhood networks) showing the correspondence (e.g., shared genes) between the two datasets, and the intra-links showing the co-expression relationships. **d** Clustering aligned networks to reveal functional links between gene modules: The multilayer network is then clustered into modules, which have the following major types: (1) the conserved modules mainly consisting of the same or orthologous genes; (2) the condition-specific modules mainly containing genes from one network; (3) the cross-network linked modules consisting of different gene sets from each network and limited shared/orthologous genes


ManiNetCluster is a novel computational method exploiting manifold learning for the comparative analysis of gene networks, enabling their comparative analysis in addition to discovery of putative functional links between the two datasets (Fig. 1, Algorithm 1). By inputting two gene expression datasets (e.g., comparing different experimental environmental conditions, different phenotypes or states), the tool constructs the gene neighborhood network for each of those states, in which each gene is connected to its top *k* nearest neighbors (i.e., genes) if the similarity of their expression profiles for the state is high (i.e., co-expression). The gene networks can be interconnected using the same genes (if the datasets are derived from two different conditions in the same organism) or orthologs (if the comparison is between two different organisms). Secondly, ManiNetCluster uses manifold alignment [17, 18] or warping [19] to align gene networks (i.e., in order to match their manifold structures (typically local and non-linear across time points), and assembles these aligned networks into a multilayer network (Fig. 1c). Specifically, this alignment step projects two gene networks, which are constructed from gene expression profiles as above, into a common lower dimensional space on which the Euclidean distances between genes preserve the geodesic distances that have been used as a metric to detect manifolds embedded in the original high-dimensional ambient space [31]. Finally, ManiNetCluster clusters this multilayer network into a number of cross-network gene modules. The resulting ManiNetCluster gene modules can be characterized into: (1) the conserved modules mainly consisting of the same or orthologous genes; (2) the condition-specific modules mainly containing genes from one network; (3) the cross-network linked modules consisting of different gene sets from each network and limited shared/orthologous genes (Fig. 1). We refer to the latter module type as the “functional linkage” module. This module type demonstrates that different gene sets across two different conditions can be still clustered together by ManiNetCluster, suggesting that the cross-condition functions can be linked by a limited number of shared genes. Consequently, and more specifically, these shared genes are putatively involved in two functions in different conditions. These functional linkage modules thus provide potential novel insights on how various molecular functions interact across conditions such as different time stages during development.

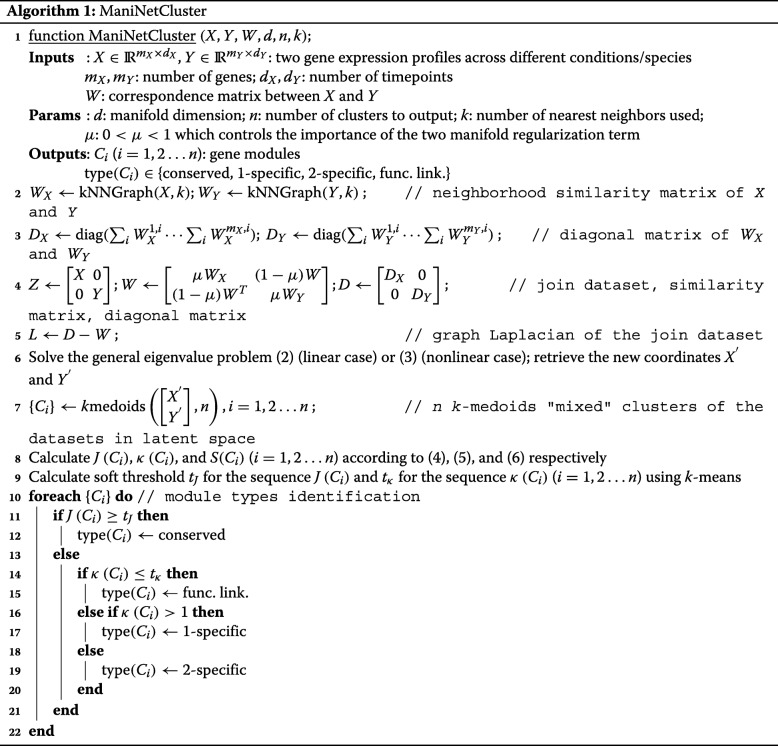


A detailed overview of ManiNetCluster is depicted in Algorithm 1. Step 1 is problem formulation. The next steps describe the primary method, which can be divided into two main parts: steps 2 to 6 are for manifold alignment; steps 7 to 22 are for the simultaneous clustering and module type identification. Our method is as follows: first, we project the two networks into a common manifold which preserves the local similarity within each network, and which minimizes the distance between two different networks. Then, we cluster those networks simultaneously based on the distances in the common manifold. Although there are some approaches that use manifold alignment in biological data [32, 33], our approach is unique since it deals with time series data (when using manifold warping) and the criteria that lead to the discovery of four different types of functional modules. The details of the two main parts are as follows.

### Manifold alignment/warping

The first steps of our method (steps 2 to 6) are based on manifold alignment [18] and manifold warping [19]. This approach is based on the manifold hypothesis and describes how the original high-dimensional dataset actually lies on a lower dimensional manifold, which is embedded in the original high-dimensional space [34]. Using ManiNetClusterwe project the two networks into a common manifold which preserves the local similarity within each network and which minimizes the distance between the different networks.

We take the view of manifold alignment [18] as a multi-view representation learning [35], in which the two related datasets are represented in a common latent space to show the correspondence between the two and to serve as an intermediate step for further analysis, e.g., clustering. In general, given two disparate gene expression profiles $X=\left \{ x_{i}\right \}_{i=1}^{m_{X}}$ and $Y=\left \{ y_{j}\right \}_{j=1}^{m_{Y}}$ where $x_{i}\in \mathbb {R}^{d_{X}}$ and $y_{j}\in \mathbb {R}^{d_{Y}}$ are genes, and the partial correspondences between genes in *X* and *Y*, encoded in matrix $W\in \mathbb {R}^{m_{X}\times m_{Y}}$, we want to learn the two mappings *f* and *g* that maps *x*_*i*_,*y*_*j*_ to $f\left (x_{i}\right),g(y_{j})\in \mathbb {R}^{d}$ respectively in a latent manifold with dimension *d*≪*min*(*d*_*X*_,*d*_*Y*_) which preserves local geometry of *X*,*Y* and which matches genes in correspondence. We then apply the framework in vector-valued reproducing kernel Hilbert spaces [36, 37] and reformulate the problem as follows to show that manifold alignment can also be interpreted as manifold co-regularization [38].

Let *f*=[*f*_1_…*f*_*d*_] and *g*=[*g*_1_…*g*_*d*_] be components of the two $\mathbb {R}^{d}$-value function $f:\mathbb {R}^{d_{X}}\rightarrow \mathbb {R}^{d}$ and $g:\ \mathbb {R}^{d_{Y}}\rightarrow \mathbb {R}^{d}$ respectively. We define $\Delta f\triangleq \lbrack L_{X}f_{1}\ldots L_{X}f_{d}\rbrack $ and $\Delta g\triangleq \lbrack L_{Y}g_{1}\ldots L_{Y}g_{d}\rbrack $ where *L*_*X*_ and *L*_*Y*_ are the scalar graph Laplacians of size *m*_*X*_×*m*_*X*_ and *m*_*Y*_×*m*_*Y*_ respectively. For $\mathbf {f}=\left \lbrack \left \lbrack f_{k}\left (x_{1}\right)\ldots f_{k}(x_{m_{X}})\right \rbrack ^{T}\right \rbrack _{k=1}^{d}$ and $\mathbf {g}=\left \lbrack \left \lbrack g_{k}\left (y_{1}\right)\ldots g_{k}(y_{m_{Y}})\right \rbrack ^{T}\right \rbrack _{k=1}^{d}$, we have $\left \langle \mathbf {f},\Delta _{X}\mathbf {f}\right \rangle _{\mathbb {R}^{dm_{X}}}=trace(\mathbf {f}^{T}L_{X}\mathbf {f)}$ and $\left \langle \mathbf {g},\Delta _{Y}\mathbf {g}\right \rangle _{\mathbb {R}^{dm_{Y}}}=trace(\mathbf {g}^{T}L_{Y}\mathbf {g)}$. Then, the formulation for manifold alignment is to solve,

1 $$ \begin{aligned} f^{*},g^{*} = \underset{f,g}{\arg\min}\; & (1-\mu)\sum_{i=1}^{m_{X}}{\sum_{j=1}^{m_{Y}}{\left\Vert {f(x}_{i})-{g(y}_{j})\right\Vert_{2}^{2}W^{i,j}}}\\ &+ \mu \left\langle \mathbf{f},\Delta_{X}\mathbf{f}\right\rangle_{\mathbb{R}^{dm_{X}}}+\mu\left\langle \mathbf{g},\Delta_{Y}\mathbf{g}\right\rangle_{\mathbb{R}^{dm_{Y}}} \end{aligned}  $$

The first term of the equation is for obtaining the similarity between corresponding genes across datasets; the second and third terms are regularizers preserving the smoothness (or the local similarity) of the two manifolds. The parameter *μ* in the equation constitutes the trade-off between preserving correspondence across datasets and preserving the intrinsic geometry of each dataset. Here, we set $\mu =\frac {1}{2}$.

As Laplacians provide intrinsic measurement of data-dependent smoothness, i.e., $\left \langle \mathbf {f},\Delta _{X}\mathbf {f}\right \rangle = \sum _{i,j}\left \Vert f(x_{i})- f(x_{j})\right \Vert ^{2}W_{X}^{i,j}$ and $\left \langle \mathbf {g},\Delta _{Y}\mathbf {g}\right \rangle = \sum _{i,j}\left \Vert g(y_{i})-g(y_{j})\right \Vert ^{2}W_{Y}^{i,j}$ the loss function in equation (1) can be rewritten as,

$$\begin{aligned} l(f,g) =& \underset{f,g}{\arg\min}\; (1-\mu)\sum_{i=1}^{m_{X}}{\sum_{j=1}^{m_{Y}}{\left\Vert {f(x}_{i})-{g(y}_{j})\right\Vert_{2}^{2}W^{i,j}}}\\ &+ \mu \sum_{i=1}^{m_{X}}{\sum_{j=1}^{m_{Y}}{\left\Vert {f(x}_{i})-{f(x}_{j})\right\Vert_{2}^{2}W_{X}^{i,j}}}\\ &+\mu\sum_{i=1}^{m_{X}}{\sum_{j=1}^{m_{Y}}{\left\Vert {g(y}_{i})-{g(y}_{j})\right\Vert_{2}^{2}W_{Y}^{i,j}}} \end{aligned} $$

Combining *W*_*X*_,*W*_*Y*_,*W* into a joint similarity matrix $W \leftarrow \left [\begin {array}{ll} \mu W_{X} & (1-\mu) W\\ (1-\mu) W^{T} & \mu W_{Y} \end {array}\right ]$ and **f**,**g** into $P=\left [\begin {array}{l}\mathbf {f}\\ \mathbf {g} \end {array}\right ]$, we have,

$$\begin{array}{*{20}l} l(f,g) = l(P) &= \sum_{i,j}\left \| P(i,\cdot)-P(j,\cdot) \right \|^{2}W^{i,j}\\ &= \sum_{i,j}\sum_{k}\left (P(i,k)-P(j,k) \right)^{2}W^{i,j}\\ &=\sum_{k} trace(P(\cdot,k)^{T}LP(\cdot,k))\\ &=trace(P^{T}LP) \end{array} $$

where *L* is the joint Laplacian of the joint dataset. We also need to add the constraint *P*^*T*^*DP*=*I*, where *D* is the diagonal matrix of *W* and *I* is the *d*×*d* identity matrix, to ignore the mapping of all instances into the subspace with dimension zero. Now, forming the Lagrange function $\mathcal {L}(P,\Lambda) = trace(P^{T}LP) + trace(\Lambda (I - P^{T}DP))$, where *Λ*=*diag*(*λ*_*i*_) is the diagonal matrix of Lagrange multipliers, and solving for the stationary points, we have *Lp*_*i*_=*λDp*_*i*_.

Thus, in parametric approach, finding minimizers *f*^∗^ and *g*^∗^ is equivalent to finding the solution of the general eigenvalue problem,

2 $$ Z^{T}{LZ}p_{i}=\lambda Z^{T}{DZ}p_{i}  $$

where $P=\lbrack p_{1},p_{2}\ldots p_{d}\rbrack =\left [\begin {array}{l}F\\ G \end {array}\right ]$ and *XF*=**f**,*YG*=**g**. Manifold alignment can also be non-parametric where, instead of finding linear form of transformation *F* and *G*, we find the new coordinates *X*^′^ and *Y*^′^ directly by solving the general eigenvalue problem,

3 $$ Lp_{i}={\lambda D}p_{i}  $$

where $\phantom {\dot {i}\!}P=\lbrack p_{1},p_{2}\ldots \ p_{d}\rbrack =\left [\begin {array}{l}X'\\ Y' \end {array}\right ]$ and *X*^′^=**f**,*Y*^′^=**g**. In both cases, the transformed datasets *X*^′^,*Y*^′^ are equal to **f**,**g** respectively.

In biological settings, the two disparate datasets *X*, *Y* share the similar underlying manifold representation because they are gene expressions from different conditions yet of the same species, or in other case, from different species yet of the same branch of evolutionary tree. From these two gene expression profiles, two gene co-expression neighborhood networks are implicitly constructed as approximations of the two manifolds. Then, the two manifolds are aligned providing the pairwise correspondence between the two datasets *W* according to the optimization problem in Eq. 1. The correspondence matrix *W* could be an identity matrix if the problem is cross-condition analysis within a specific species or could be the one whose elements $W^{i,j}=\left \{\begin {array}{cc}1 & \text {if}\ X_{i}\ \text {and}\ Y_{j}\ \text {are}\ \text {orthologous}\ \text {genes}\\ 0 & \text {otherwise} \end {array}\right.$if the problem is cross-species analysis. Alternatively, in manifold warping [19], the correspondence matrix *W* is not provided but learned with time warping function. As a result, this gives us two transformed datasets where the pairwise distance among the two dataset is diminished (compared to the original dataset).

### Simultaneous clustering and characterization of gene module types

Our ultimate goal is to simultaneously cluster the genes across different conditions so that we can actively detect which modules are conserved, which modules are specific and most importantly, which modules are functional linkage. To obtain such results, we deal with two challenges, which are (1) to integrate data across different conditions in a meaningful way and (2) to come up with a suitable distance measurement. Using manifold alignment/warping methods, we could solve those two problems together, since in manifold alignment the two datasets are projected into the latent common space where distances between corresponding points are minimized and where the locality could be measured using Euclidean distance. Thus, we perform the clustering on top of the transformed data, in which the transformation is calculated in the previous step using manifold alignment/warping methods. We applied *k-*medoids clustering for the robustness over outliers and obtained the modules whose genes might be of either of the two original networks; the proportion of such genes between networks inside a module would tell the type of that module: conserved, condition 1-specific, condition 2-specific, or functional linkage.

Simultaneously clustering is performed over the concatenation of transformed datasets: Two disparate datasets are embedded in a common latent manifold whose geodesic distances between points are preserved. The concatenation of the embedded datasets $\left [\begin {array}{c}X'\\ Y' \end {array}\right ]$ are then simultaneously clustered (using *k-*medoids). The clustering is shown in step 7 of the Algorithm 1.

We then identified two criteria to delineate the four types of genomic functional modules, which are conserved modules, data 1 specific modules, data 2 specific modules, and functional linkage modules: (1) the so-called Condition number, which is the fraction between number of genes from dataset 1 over the number of genes from dataset 2, and (2) the so-called intra-module Jaccard similarity between the two gene sets from the two conditions to be comparatively analyzed in the experimental design (e.g., phenotypes, conditions or organisms as defined by the user).

The clustering results *C*_1_,*C*_2_…*C*_*n*_ (gene modules) are of 4 types, characterized by intra-module Jaccard similarity,

4 $$ J\left(C_{i}\right)=\frac{\left|X_{i}^{'}\cap Y_{i}^{'}\right|}{\left|X_{i}^{'}\cup Y_{i}^{'}\right|}  $$

and Condition number,

5 $$ \kappa\left(C_{i}\right)=\frac{\left|X_{i}^{'}\right|}{\left|Y_{i}^{'}\right|}  $$

If *J*(*C*_*i*_) is higher than a chosen threshold, module *C*_*i*_ is a conserved module, if *J*(*C*_*i*_) is lower than the chosen threshold, we then consider the Condition number *κ*(*C*_*i*_):

- if *κ*(*C*_*i*_)≈1,*C*_*i*_ is a functional linkage module
- if *κ*(*C*_*i*_)≪1,*C*_*i*_ is a data 2 specific module
- if *κ*(*C*_*i*_)≫1,*C*_*i*_ is a data 1 specific module

Using these two criteria, a module can be determined to be a functional linkage module by **functional linkage score** *S*(*C*_*i*_),

6 $$ S(C_{i}) = 1-\frac{\left(\frac{\left|1-\kappa\left(C_{i}\right)\right|}{\max_{i}\kappa\left(C_{i}\right)} + \frac{J\left(C_{i}\right)}{\max_{i}J\left(C_{i}\right)}\right)}{\max_{i}\left(\frac{\left|1-\kappa\left(C_{i}\right)\right|}{\max_{i}\kappa\left(C_{i}\right)} + \frac{J\left(C_{i}\right)}{\max_{i}J\left(C_{i}\right)}\right)}  $$

The higher *S*(*C*_*i*_) is, the more functional linked *C*_*i*_ gets. We did not use fixed thresholds to distinguish large and small scores since these values depend on the distribution of the input datasets. Instead, we approached the threshold problem as clustering a vector data into two clusters. Thus, we employed *k*-means to implicitly determine the threshold value separating the high and low scores.

The Jaccard similarity of a module measures the degree to which the modular genes correspond to each other if they are from different datasets; e.g., the number of overlapped genes or orthologous genes. As determined by the functional linkage score (above), the functional linkage modules have a relatively low Jaccard similarity, compared to the relatively high Jaccard similarity in the conserved modules. This implies that the genes of functional linkages modules do not have high correspondence; i.e., they do not have many overlapped genes between the two compared datasets. However, ManiNetCluster clusters genes based on their Euclidean distances on a low-dimensional latent common space, which preserves their local manifold nonlinear relationships on original high-dimensional gene expression data (i.e., local, nonlinear co-expression). Thus, the genes clustered together in a functional linkage module suggest that various functions in which these genes are involved are highly likely related to each other.

### Choice of parameters

There are three parameters in the algorithms: *n*, the number of clusters (modules); *k*, the number of nearest neighbors in neighborhood graph construction; *d*, the dimension of manifold.

- The parameter *n*, indicating the number of clusters, is tunable by parameterized clustering methods such as k-means or, in our case, k-medoids. Although computational methods such as silhouette [39] or elbow [40] can be used to determine *n*, here we relied upon biological significance of modules, i.e., genes known to co-express are clustered together, to choose *n*.
- The parameter *k* influence the smoothness of the manifold constructed from data: the higher value of *k*, the smoother manifold constructed. If *k* is too small, the neighborhood graph can be sensitive to data noise; whereas, large *k* indicates the dominant of global structure over the local structure, making the approximated manifold inaccurate.
- The parameter *d* depends on the using purpose of the algorithm; for example, *d* can be set to 2 or 3 for the visualization purpose. Yet, a good practice is to choose a relatively small value of *d* since ManiNetCluster is a dimension reduction method worked by recovering a submanifold with very low dimension compared to ambient dimension of the original space.

## Results

### Datasets

To validate our methods, we applied ManiNetCluster to several previously published datasets:

1. *Developmental gene expression datasets for worm and fly:* The dataset describes time-series gene expression profiles of *Caenorhabditis elegans* (worm) and *Drosophila melanogaster* (fly), taken during embryogenesis developmental stage. The data is from the comparative modENCODE Functional Genomics Resource [41]. We took 20377 genes over 25 stages for worm and 13623 genes over 12 timepoints for fly. After removing low expressed genes (FPKM <1), we were left with 18555 and 11265 genes for worm and fly respectively. From these genes, we took 1882 fly genes and 1925 worm genes which have orthologous as correspondence information for our alignment methods [41]. The gene expression data per time stage is then normalized to unit norm.
2. *Time-series gene expression datasets for alga:* This dataset, from a previously published time series RNA-seq experiment [42], describes the transcriptome in a synchronized microalgal culturegrown over a 24hr period [42]. The data contains 17737 genes over 13 timepoints sampled during the light period and 15 timepoints sampled during the dark period. To remove technical noise, we filtered 42 genes whose expression value was less than 1 across all time points, and then log2-transformed the gene expression data. Also, we detected the outliers in the datasets by hierarchical clustering across all time points. The gene expression data per time point is then normalized to unit norm.

### ManiNetCluster reveals conserved manifold structures between cross-species gene networks

In addition to being able to cluster co-expressed genes, a unique aspect of ManiNetCluster is the ability to directly identify which modules are conserved, specific, putatively functionally linked without further analysis. ManiNetCluster organizes genes into clustered modules using a manifold alignment/warping approach. Unlike other hierarchical or *k-*means methods for clustering, our platform enables the simultaneous clustering of different datasets, offering the possibility of novel biological insight via the comparison of multiple independent experiments. This is due to the simultaneous clustering of datasets, whereas other clustering methods treat each gene expression dataset derived under different conditions separately. This uniquely allows for the identification of groups of genes, potentially linked biologically, that would otherwise be missed, possibly elucidating novel phenomena or functional inferences.

**Fig. 2:**
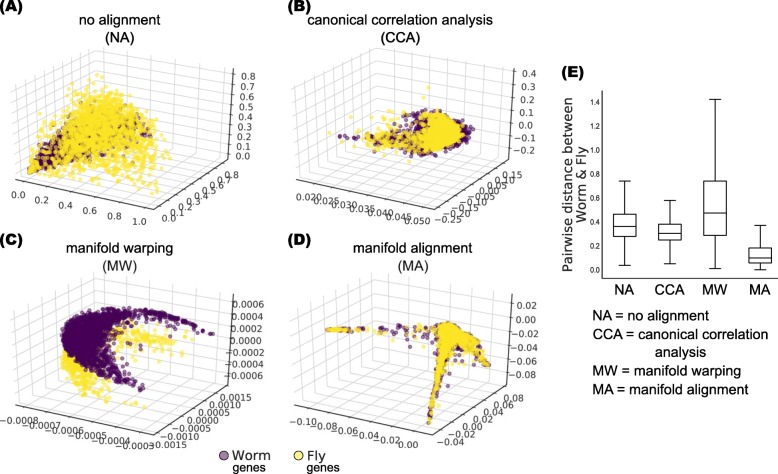
ManiNetCluster outperforms alternative methods to align cross-species developmental gene networks. **a**-**d** Scatter plots show worm and fly orthologous genes on common 3D manifolds: NA - Absence of data alignment, CCA - canonical correlation analysis, MW - manifold warping and MA - manifold alignment. **e** Boxplots show the orthologous gene distance (Chebyshev distance) on **a**-**d**. The box extends from the lower to upper quartile values of the data (pairwise distance between worm and fly), with a line at the median. The whiskers extend from the box to show the range of the data. Outliers beyond the whiskers are omitted from the plot

We previously demonstrated that orthologs across multiple species function similarly in development by using a networking approach [13, 41]. However, not all orthologs have correlated developmental gene expression profiles [26], suggesting that they may have non-linear relationships in terms of gene expression. To investigate this discrepancy, we applied ManiNetCluster to the time-series gene expression datasets of model organisms, *Caenorhabditis elegans* (worm) and *Drosophila melanogaster* (fly), taken during embryogenesis, to determine whether orthologous genes have non-linear relationships, and if these relationships are also conserved across species. We employed ManiNetCluster to align cross-species developmental gene networks and compared the results with other methods, including canonical correlation analysis (CCA) [43]. These analyses indicated that the orthologous genes between worm and fly are better aligned by non-linear manifold learning than the linear methods, as indicated by their distances after alignment: CCA = 632.44 vs. ManiNetCluster = 276.32 (t-test *p*-value <2.2×10^−16^) in terms of sum of pairwise distances (Fig. 2). (We use Chebyshev distance because it is a good approximation of the Euclidean distance (with less computing power) which could capture the skeleton of the data shape effectively [44].) This suggests that non-linear interactions exist between evolutionary conserved functions encoded by orthologous genes across worm and fly during development. Note that in this experiment, we set the parameter *k*, number of nearest neighbors, to be 3. We also tried other value of *k* from 1 to 7, all of them deliver good results (Additional file 2: Figure S1). The parameter *d* is set to be 3 for the visualization purpose. Other choices of *d* (i.e., *d*=2,4,6,8,10,12) are also experimented (Additional file 2: Figure S2). We found that ManiNetCluster outperforms others when *d* is small (*d*=2,4,6), which implies that it followed manifold hypothesis and revealed a very low dimensioned submanifold (compared to the high dimensioned ambient space). However, when increasing the manifold dimensions (e.g., *d*=8,10,12), the intrinsic geometry of the data cannot be retrieved due to a higher dimension space resembling the original linear space, leading ManiNetCluster working roughly equivalent to others.

### ManiNetCluster identifies putative genomic function links between cross-condition gene networks

**Fig. 3:**
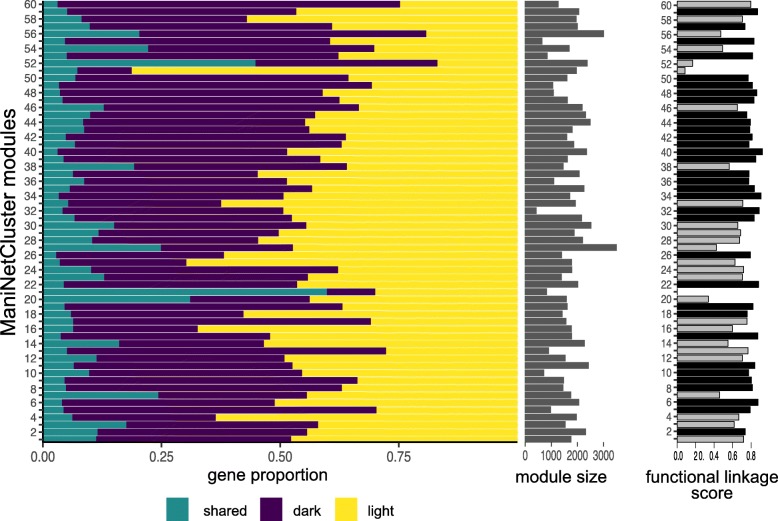
Identification of gene modules, including function links between light and dark condition in *Chlamydomonas reinhardtii*. We applied ManiNetCluster to the algal time series diurnal transcriptomes [42]. For the purposes of these analyses, the transcriptomes collected during the light period were treated as an independent experiment from those collected during the dark period. In total, we identified 60 gene modules. The proportion of each module comprised of light period specific (yellow), dark period specific (purple), and shared (teal) is shown. Module size is indicated on the right of the modules. Further on the right are functional linkage scores; high scores (highlighted in black) indicate functional linkage modules. (See Tables S1 and S2 for details of all modules)

As a case study to demonstrate the uniqueness and validity of ManiNetCluster for comparing between conditions, we used a previously published dataset [42]. This dataset describes the transcriptomic dynamics of a synchronized microalgal culture grown over a 24hr period, and was specifically chosen to test ManiNetCluster due to the comprehensiveness of the time series (samples taken at 1 h or 30 min intervals over two independent 24 hour periods [42]). Using the ManiNetCluster algorithm we delineated the transcriptomes sampled during the light period vs. the dark period of the 24 h experiment. After alignment (in which ManiNetCluster again outperformed CCA: ManiNetCluster = 128.00 vs. CCA = 713.50 in terms of sum of pairwise distances (t-test *p*-value <2.2×10^−16^)), we simultaneously clustered the two groups of transcriptomes, treating the light- and dark-collected samples as independent experiments. ManiNetCluster clustered the two datasets (i.e., light period and dark period) into 60 modules of *Chlamydomonas reinhardtii*, and delineated the genes in each into light-specific, dark-specific and shared between light and dark (Fig. 3; Tables S1 and S2). Based on the metrics (intra-module Jaccard similarity, Condition number) that quantify relative light/dark gene proportions (Methods; Additional file 1: Table S2), we detected four types of module: conserved, light or dark specific, and functionally linked. The functional linkage modules consist of different gene sets from light and dark networks with very limited shared genes (Additional file 1: Table S2). For example, Module 60 is a dark-specific module due to a high proportion of dark period genes and Module 21 is a conserved module since it has a high fraction of shared genes (functional linkage score = 0.000)(Fig. 3; Tables S1 and S2). Module 34 is a functional linkage module since it contains a low proportion of shared genes and high proportion of different light and dark period genes (functional linkage score = 0.909) (Fig. 3; Additional file 1: Tables S1 and S2). Many modules are highly enriched for genes expressed during the light period, the dark period and for shared in both the light and dark networks. This is clearly demonstrated in Modules 34, 52 and 60, which are enriched for shared, light and dark genes respectively (Figs. 3 and. 4; Additional file 1: Tables S1 and S2). These groupings indicate that the proteins encoded by genes in these modules could have related specific roles in either light-, dark- or both light and dark-specific metabolism. Consequently, the gene sets within each module could be used to provide functional inferences for each gene and the co-expressed genes across the module. For example, Module 21 is highly enriched for genes encoding proteins involved in protein synthesis in the light-dark shared fraction of the module, suggesting that these proteins are active in the synthesis of proteins for both the light and dark periods. Note that in this experiment, we still set the parameter *k* to be 3 and parameter *d* to be 3. The value 60 of parameter *n* is chosen because it gives us the biological interpretability of each modules as mentioned in this paragraph.

**Fig. 4:**
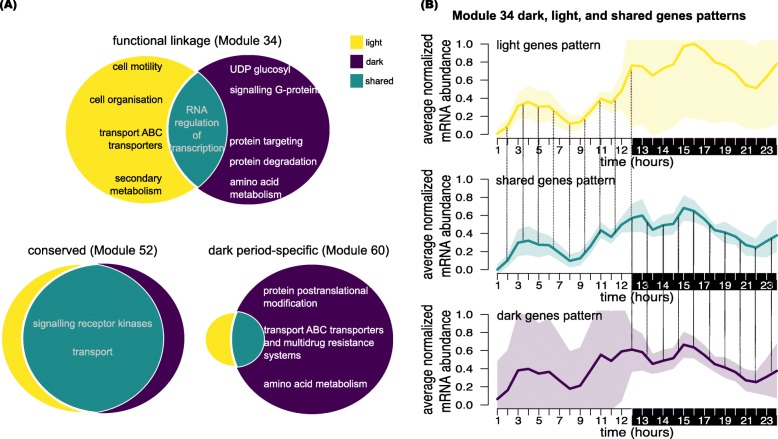
Functional linkage, conserved and condition-specific modules of *Chlamydomonas reinhardtii* between light and dark condition **a** Module types identified by ManiNetCluster, using an algal diurnal dataset [42] with light-period and dark-period transcriptomes treated as independent experiments. Example modules are shown: (1) Module 52 - a conserved module in which the proportion of shared genes is high; (2) Module 60 - a dark specific module in which the proportion of dark period genes is high; (3) Module 34 - a functional linkage module in which the proportion of shared genes is low and the proportion of light period genes and dark period genes are approximately equal. Functional enrichment for each were generated using MapMan (a tool for functional annotation based on gene ontologies designed for photosynthetic organisms) [46]. **b** Expression patterns of example functionally linked modules: Expression patterns of light, dark, and shared genes of module 34 are shown. The shared genes (shown in teal) correlate with light genes (yellow) in light condition (13 first time points) and with dark genes (purple) in dark condition (15 last time points) as indicated by vertical dashed lines. Note that the dark genes in light condition and the light genes in dark condition are not identified as the error bar (light purple shading in 13 first time points and light yellow shading in 15 last time points) are too large; this indicates that the shared genes serve as a bridge connecting the gene expression from light to dark conditions. The light and dark periods are shown with shading on the x axis. Complete module data are in Tables S1 and S2

To further investigate and validate the functional linkage modules, we focus here specifically on two Modules, 6 and 34 (Figs. 3 and. 4; Tables S1 and S2). These modules were chosen as examples since they both exhibit low intra-module Jaccard similarities (0.04 and 0.03 for Modules 6 and 34 respectively) and their Condition number values is approximately 1 (1.13 and 1.04 for Modules 6 and 34 respectively), indicative of a small number of shared genes and similar numbers of light and dark period genes (Additional file 1: Table S2); in short, their functional linkage scores are 0.876 and 0.909 respectively. Module 34 contains a total of 598 genes. Of these, the mRNA abundance of 284 genes within the module are from the light period and 295 are from the dark period (Figs. 3 and. 4; Additional file 1: Table S1). Of those genes annotated, the light period genes are functionally enriched for flagellar associated proteins (FAPs [45]), the cell motility and cell organization Mapman ontologies [46] and the dark period genes contain a number of transporters, Greencut associated genes [47–49] and genes encoding proteins involved in DNA synthesis. More notably, 19 genes are shared between the light and dark periods, meaning that these genes tightly co-express with both the light genes during the light period and the dark genes during the dark period (Fig. 4; Additional file 1: Table S1). These 19 genes encode proteins functionally enriched for aspects of regulation, including protein post-translational modification and RNA regulation (8 of the 19 genes have an associated gene ontology, all of which are related to regulation. These ontologies (and gene annotations where they exist), together with the interactions with the rest of the module, suggest the possibility of a hierarchical gene/protein regulatory network, with these genes putatively imposing some aspect of regulation upon the rest of the module. Similarly, Module 6 contains 721 genes, of which 326 are dark-period specific, 368 are light-period specific and 27 are shared. Again, these 27 are enriched for genes encoding proteins with putative regulatory roles (Fig. 4; Additional file 1: Table S1). Additional modules that display the same statistical characteristics are Modules 15 and 40 (as indicated by the intra-module Jaccard similarities and Condition numbers and functional linkage scores; Fig. 4, Additional file 1: Table S2).

## Discussion

### ManiNetCluster clusters genes into modules in a comparable manner to other methods

To test the validity of the modules generated by ManiNetCluster, we scrutinised each cluster from a biological perspective by confirming their consistency with previous experimental findings [42]. In that study, using the *k-*means algorithm, 12,592 genes were clustered into co-expressed modules. Since this number represents >70*%* of the genes on this organism’s genome, we reasoned such a significant number would provide an appropriate testbed for corroborating our method described here. The two methods of module generation performed on the same original dataset are highly similar, indicating the general validity of the ManiNetCluster approach in terms of biological significance. Firstly, there is a high degree of similarity of co-clustered genes between modules generated using ManiNetCluster and the *k-*means method (ARI = 0.95 and 0.95 for light and dark period modules respectively). Secondly, genes encoding proteins of related function are co-expressed, since interacting proteins are required together and under the same conditions.

Analysis of the modules generated by ManiNetCluster indicates functionally-related genes are co-clustered, as expected. For example, the genes encoding proteins constituting the photosynthetic complexes LHCI, LHCII, PSI, PSII, *b*_6_ *f* and the chloroplast ATP synthase are nearly entirely contained within the ManiNetCluster Modules 20 and 21 (Additional file 1: Table S1). Equally, the genes encoding subunits of the mitochondrial respiratory complexes are almost entirely contained within two modules (Additional file 1: Table S1), as are the genes encoding many other functionally-related proteins (Additional file 1: Table S1). Together, these two analyses serve to confirm the veracity of our method for clustering similarly expressed genes.

### Comparison of maniNetCluster vs. other clustering methods

**Fig. 5:**
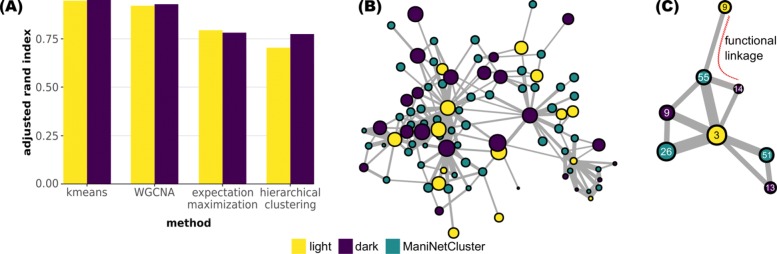
Comparison of ManiNetCluster with other clustering methods. **a** The adjusted rand index between ManiNetCluter clustering and other methods, as shown, indicates ManiNetCluster is consistent with *k-*means and WGCNA but less so with expectation maximization and hierarchical clustering. **b** comparison of 60 cross-condition modules detected by ManiNetCluster as well as 34 light period modules and 30 dark period modules separately detected by WGCNA by constructing a network, consisting all ManiNetCluster and WGCNA modules as nodes. The links between two nodes indicate the genes shared by both modules. Node size indicates the degree of that node. Links with very low weight are omitted. The triad of the network among three different kinds of nodes (i.e., ManiNetCluster module, WGCNA “light-period” module and WGCNA “dark-period” module) indicates the functional linkage type of an ManiNetCluster module. An open triad patterns indicates a functional linkage module. **c** Subgraph of the network in **b** demonstrating a functional linkage module (Module 55). The subgraph also identifies a putative functional link between two WGCNA modules, Light-Module 9 and Dark-Module 14

Finally, we compared ManiNetCluster to the state-of-the-art methods, including WGCNA, *k-*means, Hierarchical Clustering (HC), Expectation Maximization (EM) that cluster individual gene networks into modules to evaluate the consistency of our clustering. (The technical details of these other methods are specified in Additional file 2) As a measure of evaluation, we employed the adjusted rand index (ARI) to assess the overlap of gene modules from these other methods (Fig. 5). Specifically, the similarity between two data clusterings *C*={*C*_1_,*C*_2_…*C*_*k*_} and $C^{'}=\{C_{1}^{'},C_{2}^{'}\ldots C_{l}^{'}\}$ is computed using the adjusted rand index (ARI) as follows:

$$R_{\text{adj}}\left(C,C^{'}\right)=\frac{\sum_{i=1}^{k}{\sum_{j=1}^{l}\left(\begin{array}{c}m_{\text{ij}}\\ 2 \end{array}\right)-t_{3}}}{\frac{1}{2}(t_{1}+t_{2})-t_{3}} $$

where $t_{1}=\sum _{i=1}^{k}\left (\begin {array}{c}\left |C_{i}\right |\\ 2 \end {array}\right),\ t_{2}=\sum _{j=1}^{l}\left (\begin {array}{c}\left |C_{j}^{'}\right |\\ 2 \end {array}\right),\ t_{3}=\frac {2t_{1}t_{2}}{n(n-1)}, m_{\text {ij}}=\left |C_{i}\cap C_{j}^{'}\right | $, and *n* is the number of observations (i.e., genes). The value of this index is ranged from 0 (independant clusterings) to 1 (identical clustering). For this assessment, we again used the datasets from a previously published time series RNA-seq experiment [42]. Using this data, we found that in general, the ManiNetCluster modules overlap with those identified by other methods (e.g., WGCNA = 0.92 and 0.93, *k-*means = 0.95 and 0.95, EM = 0.81 and 0.79, HC = 0.70 and 0.78 for light and dark modules, respectively). The high value of ARI over *k-*means and WGCNA indicates that ManiNetCluster is effective (consistent to *k-*means clustering, proved to deliver meaningful biological results in previous experiment [42]) and robust (consistent to WGCNA). This demonstrates that ManiNetCluster modules are highly consistent with the state-of-art methods in terms of clustering the genes using each condition’s dataset, but more importantly, since ManiNetCluster modules also include the genes across conditions, they provide additional insights into the connections among various genomic functions across different conditions whereas the state-of-art methods do not.

However, though these state-of-art methods find the modules from individual conditions (e.g., WGCNA light modules, dark modules), we can still use ManiNetCluster modules to link their modules for uncovering additional potential cross-condition links. To demonstrate this capability, we compared the ManiNetCluster modules with those collected using WGCNA to evaluate how they overlap, potentially providing additional functional linkages between WGCNA light and dark modules. Specifically, we connected the modules of WGCNA and ManiNetCluster where they share genes, and created a module network in which edge weights are the number of shared genes (Fig. 5b and c). We found that functional linkage modules generated by ManiNetCluster can connect multiple WGCNA modules (Fig. 5), i.e., two separated WGCNA modules that are potentially functional linked if seeing through the perspective of our method. We thus investigated the triad patterns (among ManiNetCluster modules, WGCNA modules for light, WGCNA modules for dark) of such network to analyze if a ManiNetCluster module is of functional linkage type, which is correspondent to the opened triangle (depicted by opened red curve) shown in Fig. 5c. For example, Module 55 contains a total of 233 genes, of which 10 are co-expressed with both the light and dark period genes across the complete 24 hour experiment (Additional file 1: Table S1). Within the 10 shared genes are FTSY, which has a demonstrated role in LHC assembly [50] suggests the possibility of additional roles during the dark period. Another gene in this group is *FDX7*, encoding a predicted uncharacterized ferrodoxin [51], suggestive of a role in both the light and dark periods for this protein also. The triad pattern shown in Fig. 5c also suggests a functional link between WGCNA Light-Module 9 and WGCNA Dark-Module 14, which cannot be detected by WGCNA itself, since they have shared genes with a ManiNetCluster functional linkage module (Module 55). We also compared ManiNetCluster, WGCNA, and *k*-means in terms of asymptotic complexity (Additional file 2: Table S3).

## Conclusions

Elucidating and understanding the data encoded within each organism’s genome remains the greatest challenge in modern biology. To help extract more information from gene expression datasets, we have developed a novel computational method, ManiNetCluster, which aims to reveal functional linkages of gene networks across conditions (e.g., species, time points). In particular, this method extends the manifold learning approaches that capture non-linear relationships among genes to simultaneously cluster different gene networks to discover cross-network gene modules linking various genomic functions together. For instance, our tool could be used interrogate two transcriptomes investigating the gene expression effects of two different drug treatments, possibly aiding in the identification of synergistic or antagonistic consequences of dual delivery. In this paper, we demonstrated ManiNetCluster for two networks; yet, it can be extended to analyze multiple networks[18].

As a tool, ManiNetCluster falls within an emerging field of research, called multi-view learning [52, 53]. Many biological datasets are naturally comprised of different representations or views, which often provide compatible and complementary information [54], e.g., light and dark period transcriptome of an alga, gene expression of worm and fly whose genes are orthologous or multi-omics single cell data [55]. It is natural to integrate these views together (in a non-linear way) prior to any analysis rather than analyzing each view separately, and then concatenating them (in a linear way). ManiNetCluster realizes a general multi-view learning approach by implementing manifold alignment/warping to combine multiple views into a common latent subspace for further analysis, i.e., clustering. Previous studies have emphasized the importance of multiview learning in heterogenous biological data [54] or discussed different methods realizing multiview learning [52, 53] but, to the best of our knowledge, very few of them [55, 56] regarded manifold alignment as such a method. In our approach, manifold alignment is considered to be a natural and effective method for multiview representation learning.

ManiNetCluster can be used as general purpose to study other biological networks with additional linkage types such as protein-protein interactions. One possible application is the single cell. Increasing single cell data enable identification of interactions among various cell types and seeing how cell types contribute to the phenotypes at the tissue level such as tissue gene expression. Moreover, nonlinearity has been found to widely exist among cell interactions. Thus, ones can also apply this method to single cell gene networks and find out the genomic functional linkages across cell types, providing potential novel insights on cell type interactions.

## Supplementary information

**Additional file 1** Tables, including: **Table S1** Module data generated by ManiNetCluster. **Table S2** ManiNetCluster module statistics.

**Additional file 2** Additional_methods_table_sand_figures, including: Methods for the construction of WGCNA and other clustering techniques. **Table S3** Complexity of ManiNetCluster, WGCNA, and *k*-means. **Figure S1** ManiNetCluster performs best with small values of *k*. **Figure S2** ManiNetCluster performs best with small values of *d*. **Figure S3** Characterization of module types according to Jaccard indices and Condition number. **Figure S4** Cross-heatmap demonstrating the relationship between modules. **Figure S5** Expression patterns of example functionally linked modules.

**Additional file 3** Tutorial, including: ManiNetCluster Tutorial.

## Data Availability

All data generated or analysed during this study were included in this published article and the additional files.

## Abbreviations

ARI: Adjusted rand index
CCA: Canonical correlation analysis
EM: Expectation maximization
FAP: Flagellar associated proteins
FPKM: Fragments per kilobase million
HC: Hierarchical clustering
kNNGraph: K-nearest neighbor graph
S^3^: Symmetric substructure score
WGCNA: Weighted gene co-expression network analysis
