# Supplementary material for: ManiNetCluster: a novel manifold learning approach to reveal the functional links between gene networks
Source: BMC Genomics. 2019 Dec 30;20(Suppl 12):1003. doi: 10.1186/s12864-019-6329-2 (PMC6936142; doi:10.1186/s12864-019-6329-2)
Supplement: Supplementary file 2 — Additional file 2 Additional_methods_table_sand_figures, including: Methods for the construction of WGCNA and other clustering techniques. Table S3 Complexity of ManiNetCluster, WGCNA, and k-means. Figure S1 ManiNetCluster performs best with small values of k. Figure S2 ManiNetCluster performs best with small values of d. Figure S3 Characterization of module types according to Jaccard indices and Condition number. Figure S4 Cross-heatmap demonstrating the relationship between modules. Figure S5 Expression patterns of example functionally linked modules. [file 12864_2019_6329_MOESM2_ESM.pdf]

SUPPLEMENTAL MATERIALS FOR

A novel manifold learning approach to reveal the functional links between gene networks

Nam D Nguyen[1], Ian K Blaby[2*] and Daifeng Wang[3,4*]

[*]Correspondences: iblaby@bnl.gov, daifeng.wang@stonybrookmedicine.edu
[1]Deparment of Computer Science, Stony Brook University, NY 11794 Stony Brook, USA
[2]Biology Deparment, Brookhaven National Laboratory, NY 11973 Upton, USA
[3]Department of Biomedical Informatics, Stony Brook University, NY 11794 Stony Brook, USA
[4]Stony Brook Cancer Center, Stony Brook Medicine, NY 11794 Stony Brook, USA

Construction of WGCNA and other clustering methods

To compare ManiNetCluster clusterings with WGCNA clustering, we also construct the weighted gene co-expression networks and clustering as follows:

We constructed the gene co-expression networks by connecting all possible gene pairs by edges whose weights are the combination of Pearson correlations and Euclidean distance of their time-series gene expression profiles. The reason for this combination is that Pearson correlations well capture the "shape" of the data (up or down of the expression) while Euclidean distance well capture the "scale" of the data (low or high of the expression). First, we constructed a similarity matrix S of a dataset X as follows [1]:

$$S = \operatorname{sgn}(\rho\,(X))\frac{|\rho(X)| + \left(1 - \frac{log(d(X)+1)}{max(\log(d(X)+1))}\right)}{2}$$

where $\rho(X)$ depicts the pairwise Pearson correlation and $d(X)$ depicts the pairwise Euclidean distance of the input dataset. The first term of the equation is the sign of the Pearson correlation, preserving the sign of the interaction. The second terms combine the Pearson correlation and the "Euclidean closeness", which is the log inverse Euclidean distance. The result S, measuring the similarity between two genes, is a number, ranging from -1 to 1, indicating the strength of correlation and its sign of interaction, i.e., positive or negative [1].

Next, we construct the adjacency matrix from the similarity matrix. We use the power transformation, as suggested by Zhang et al. [2] to reduce the number of spurious correlations in the data and to transform the network into a scale-free topology [2]. The resulted adjacency matrix $A = \{a_{\mathrm{ij}}\}$ is computed from similarity matrix $S = \{s_{\mathrm{ij}}\}$ as follows:

$$a_{\mathrm{ij}} = \left(\frac{1}{2}\left(1 + s_{\mathrm{ij}}\right)\right)^{\beta}$$

The gene co-expression networks were then clustered into modules by using the *cuttreeDynamicTree* function in WGCNA (weighted correlation network analysis) R package [3].

Clustering results of k-means, hierarchical clustering, and expectation maximization is obtained directly from functions *kmeans()*, *cutree(hclust())*, and *Mclust()*

respectively in R packages *cluster* [4] and *Mclust* [5]. These methods are not for simultaneous clustering, so we perform these on light period genes and dark period genes separately. The number of cluster (k) is 34 for light period genes and 30 for dark period genes.

Supplemental Figures and Tables

	ManiNetCluster	WGCNA	k-means
time	$O(m^2 d)$	$O(m^2 d)$	$O(nkdi)$
space	$O(m^2)$	$O(m^2)$	$O(nd + kd)$

Table S3:
Asymptotic complexity of ManiNetCluster, WGCNA, and k-means. Given m points in $\mathbb{R}^d$, MainNetCluster consists of these main steps: (1) constructing the kNNGraphs and creating joint structures Z, W, D, L as in algorithm 1, (2) solving the general eigenvalue problem, (3) clustering the transformed joint dataset. The cost of step (1) is $O(m^2 d)$ which is dominated by the constructing of adjacency matrices. Solving the eigendecomposition in step (2) requires $O(m^2 k)$ operations. The runtime complexity of step (3) is $O((m - n)^2 n)$ where n is the number of clusters. Asumming that $n, k \ll m$, the overall time complexity of ManiNetCluster is $O(m^2 d)$. WGCNA also needs three sequential steps: (1) constructing the adjacency matrices and obtaining a soft threshold, which has a complexity $O(m^2 d)$, (2) calculating TOM matrices, which also has a complexity $O(m^2 d)$ (3) hierarchical clustering, which has a complexity $O(m \log m)$. Overall, the time complexity of WGCNA is $O(m^2 d)$. Thus, the running times of ManiNetCluster and WGCNA are asymptotically the same. Their space complexities are also equal, $O(m^2)$, since the TOM matrices of WGCNA and the Laplacian of ManiNetCluster are maintained in memory. However, in practice, the memory required by ManiNetCluster can be two times of the memory required by WGCNA. This is due to the fact that WGCNA is not designed for comparative analysis and that it can be run sequentially dataset by dataset. In contrast, ManiNetCluster is used for comparative analysis, which need to store the two datasets, i.e. the joint Laplacian, at the same time, resulting in more space needed. The time and space complexity of k-means are $O(nkdi)$ and $O(nd + kd)$ respectively where the parameter i is the number of iteration used in Lloyd's algorithm. It is worth to note that, unlike k-means, the clustering results of WGCNA and ManiNetCluster are not from raw data, but from a biologically enriched representation of data, i.e. gene co-expression network in WGCNA and manifold in ManiNetCluster. Also, in contrast to ManiNetCluster, additional complicated procedures are required in WGCNA to detect the conserved or specific modules.

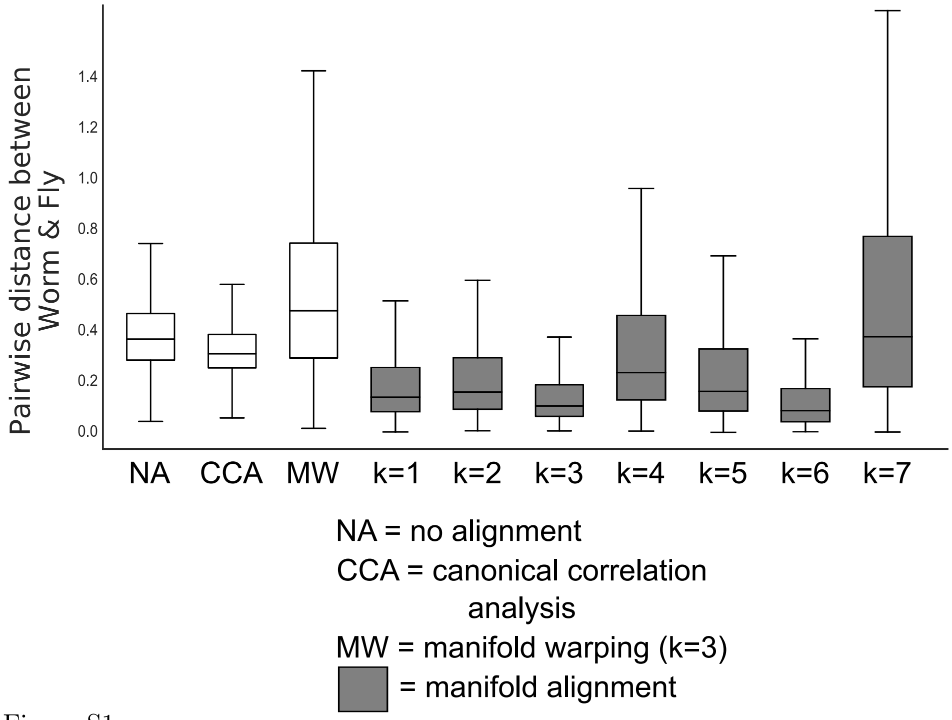

Figure S1:
ManiNetCluster performs better with small values of k. The results of manifold alignment with different parameters k (number of nearest neighbors in neighborhood network) compared to other methods. We keep the same value of dimension ($d = 3$) and experiment with values of k, ranging from 1 to 7. The results show the best performance (distances between corresponding pairs of the two species) in the case of $k = 3$ and $k = 6$

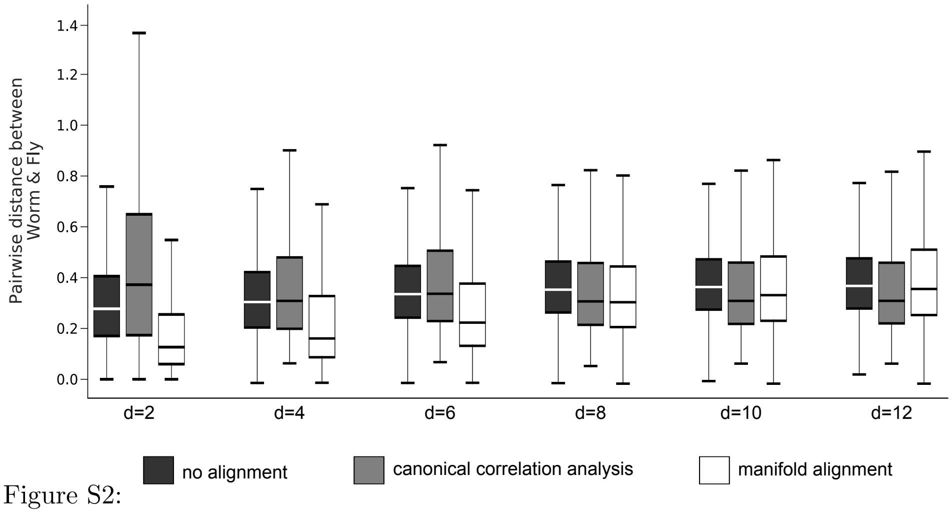

Figure S2:

ManiNetCluster performs better with small values of d. The results of manifold alignment with different parameters d (the dimension of manifold) compared to other methods. We keep the same number of nearest neighbors ($k = 3$) and experiment with values of d, ranging from 2 to 12. The results show that the lower dimension is, the better manifold alignment performs. According to the manifold hypothesis, ManiNetCluster works best with a value of d being much smaller than 12, which is the ambient dimension. In fact, with value of d being 2 or 4, the alignment results are significant better than CCA. Starting from $d = 8$, the results of ManiNetCluster are roughly similar to that of CCA since, in a higher dimension space, the intrinsic geometry of the data cannot be retrieved.

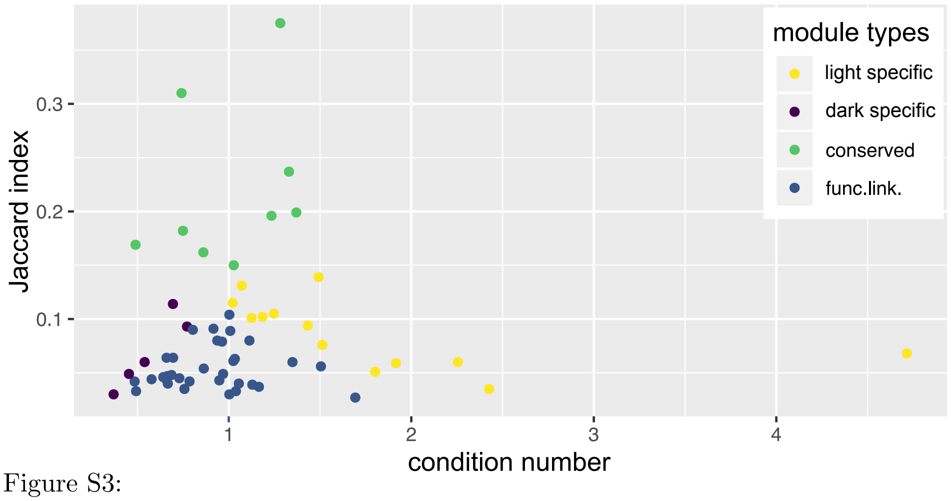

Figure S3:

Characterization of module types according to Jaccard indices and Condition number. The 60 modules in the span of Jaccard similarities and Condition number. As indicated by equation (4), (5), (6), conserved modules dominate the upper side, dark- and light specific modules are in the lower left and lower right side respectively; functional linked modules are in lower side, concentrating around where condition number is 1.

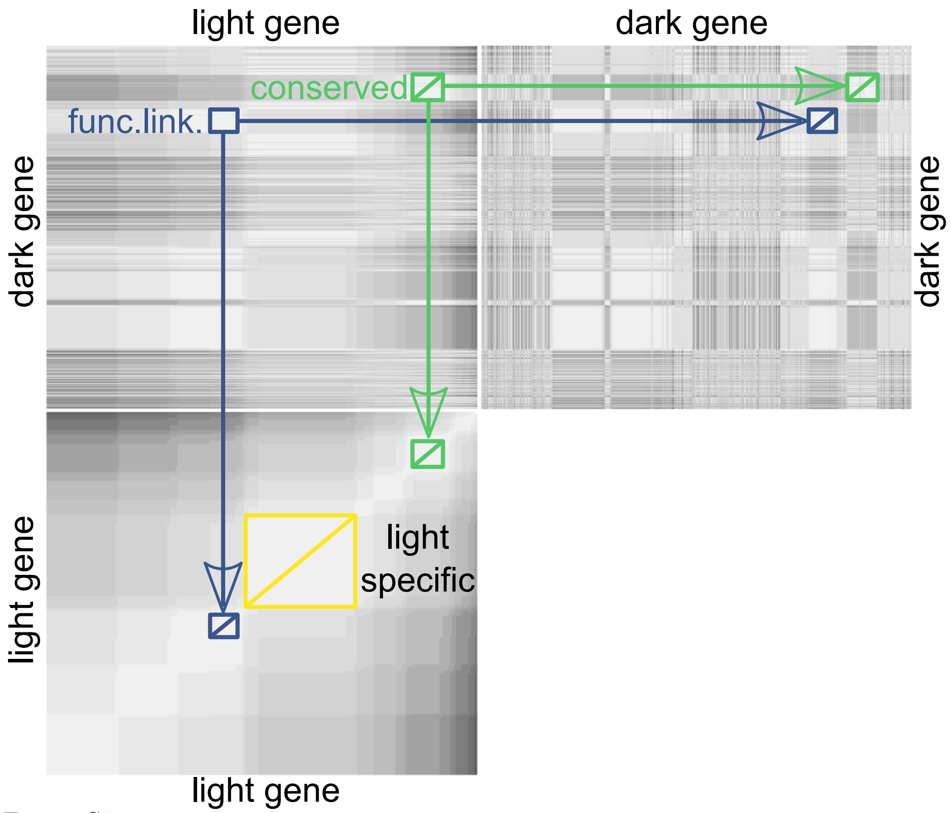

Figure S4:

Cross-heatmap demonstrating the relationship between modules in each condition (*i.e.* light period-specific or dark period-specific), which reveals the module types. The off-diagonal module (depicted in blue) which has corresponding modules in both light and dark clusters is an example of a functionally linked module, and the on-diagonal module (depicted in green) which has corresponding modules in both light and dark clusters is an example of condition-specific module.

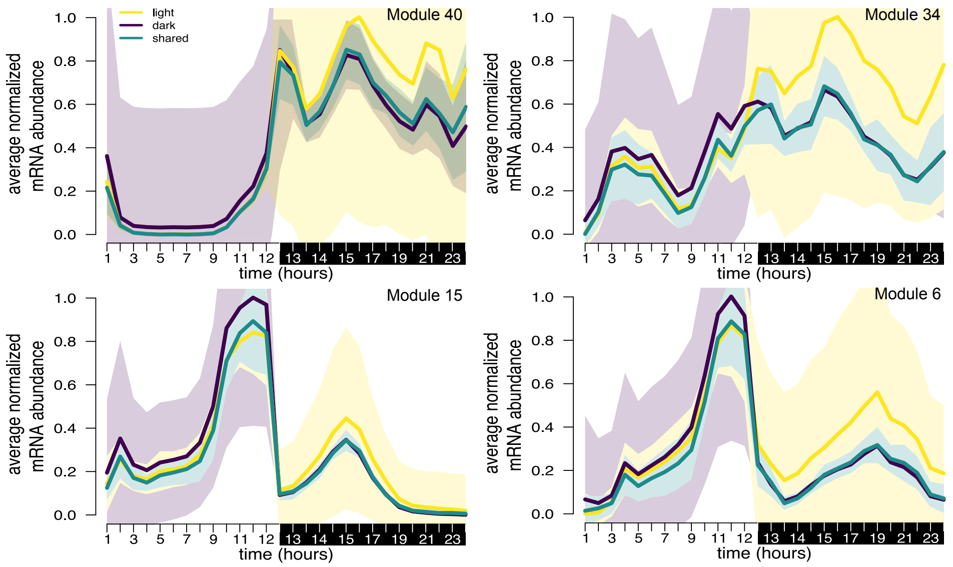

Figure S5:

Expression patterns of example functionally linked modules. Expression patterns of light, dark, and shared genes of modules 34, 6, 15, and 40 are shown.

Author details

[1]Deparment of Computer Science, Stony Brook University, NY 11794 Stony Brook, USA. [2]Biology Deparment, Brookhaven National Laboratory, NY 11973 Upton, USA. [3]Department of Biomedical Informatics, Stony Brook University, NY 11794 Stony Brook, USA. [4]Stony Brook Cancer Center, Stony Brook Medicine, NY 11794 Stony Brook, USA.

References

1. Hughitt, V.K.: Supplemental file: sandfly co-expression cluster analysis (2016)
2. Zhang, B., Horvath, S.: A general framework for weighted gene co-expression network analysis. Statistical applications in genetics and molecular biology **4**(1) (2005)
3. Langfelder, P., Horvath, S.: Wgcna: an r package for weighted correlation network analysis. BMC bioinformatics **9**(1), 559–559 (2008)
4. Maechler, M., Rousseeuw, P., Struyf, A., Hubert, M., Hornik, K.: Cluster: cluster analysis basics and extensions. R package version **1**(2), 56 (2012)
5. Scrucca, L., Fop, M., Murphy, T.B., Raftery, A.E.: mclust 5: Clustering, classification and density estimation using gaussian finite mixture models. The R journal **8**(1), 289 (2016)
