# Supplementary material for: ManiNetCluster: a novel manifold learning approach to reveal the functional links between gene networks
Source: BMC Genomics. 2019 Dec 30;20(Suppl 12):1003. doi: 10.1186/s12864-019-6329-2 (PMC6936142; doi:10.1186/s12864-019-6329-2)
Supplement: Supplementary file 3 — Additional file 3 Tutorial, including: ManiNetCluster Tutorial. [file 12864_2019_6329_MOESM3_ESM.html]

# ManiNetCluster

A novel manifold learning approach to reveal thefunctional links between gene networks

author: Nam Nguyen, Ian Blaby, Daifeng Wang

date: February 16, 2019

# Abstract

The coordination of genomic functions is a critical and complex process across biological systems such as phenotypes or states (e.g., time, disease, organism, environmental perturbation). Understanding how the complexity of genomic function relates to these states remains a challenge. To address this, we have developed a novel computational method, ManiNetCluster, which simultaneously aligns and clusters gene networks (e.g., co-expression) to systematically reveal the links of genomic function between different conditions. Specifically, ManiNetCluster employs manifold learning to uncover and match local and non-linear structures among networks, and identifies cross-network functional links. We demonstrated that ManiNetCluster better aligns the orthologous genes from their developmental expression profiles across model organisms, than state-of-the-art methods. This indicates the potential non-linear interactions of evolutionarily conserved genes across species in development. Furthermore, we applied ManiNetCluster to time series transcriptome data measured in the green alga Chlamydomonas reinhardtii, to discover the genomic functions linking various metabolic processes between the light and dark periods of a diurnally cycling culture. We identified a number of genes putatively regulating processes across each lighting regime.

The following text provides an overview of gene expression analysis workflow with ManiNetCluster describing both gene clustering and alignment. The workflow of ManiNetCluster is depicted in the figure below:

ManiNetCluster Workflow Schematic. (A) The inputs of the workflow are the two time series gene expression datasets collected from different conditions (as in this example) or from two different organisms. The number of genes and/or the number of timepoints need not be the same in each dataset. (B) From the input data, gene co-expression neighborhood networks are constructed, which approximate the manifolds where the datasets are concentrated. (C) Using manifold alignment and manifold warping methods, the two gene expression profiles are aligned across time series in a common manifold. The outcome of this step is a multilayer network consisting of two types of links: the inter-links (between the two co-expression neighborhood networks) showing the correspondence (e.g., shared genes) between the two datasets, and the intra-links showing the co-expression relationships. (D) The multilayer network is clustered into modules. Four distinct types of modules are revealed in this step: conserved modules containing high proportion of shared genes, dataset 1-specific modules containing a high proportion of genes from dataset 1, dataset 2-specific modules containing a high proportion of genes from dataset 2, and function linkage modules containing near equal numbers of genes from both datasets, which are the same gene (if conditions are compared from the same organism) or orthologs (if the organism differs between the two compared datasets).

# To begin

First you will need to download the package from github. To seamlessly integrate both Python and R in the same environment, you will need to install the reticulate package.

```
install.packages("reticulate")
```

Then, you can start by installing the devtools package from CRAN and load it. Installation should take less than one minute.

```
install.packages("devtools")
```

Now, you specify the Python library for reticulate:

```
reticulate::use_python("/Users/tkn/anaconda3/bin/python")
reticulate::py_config()
```

Finally, install ManiNetCluster and load it:

```
devtools::install_github("namtk/ManiNetCluster")
library(ManiNetCluster)
```

The following demo is provided to walk through the basic functionality of ManiNetCluster. It should take less than 10 minutes to complete on a standard desktop computer.

# Introduction

ManiNetCluster is a package that takes as input two or more gene expression profiles, and the gene correspondences (i.e. orthologs) for cross-species comparison. ManiNetCluster has 3 essential steps:

1. Construct gene co-expression networks from gene expression data
2. Align the networks incrementally in pseudo-time using manifold alignment and warping techniques
3. Cluster genes into modules of four different types (type I: conserved modules, type II, type III: specific-specific modules, type IV: functional connectivity modules).

Included in our package is an example dataset. These data are published in (1) and describe a light/dark timecourse of synchronized cultures of a green alga.

We provided here the sample data of time series gene expression data of Chlamydomonas reinhardtii. The full dataset includes more than 17000 genes but in this example, to demonstrate cross-species comparison, we have limited the data to genes which have orthologs (as determined by InParanoid8 (2)) by taking genes being orthologous to Arabidopsis thaliana. Users can find the data (in .csv format) in the github repository (namtk/ManiNetCluster/data/).

The first step is to load the data:

```
X <- as.matrix(read.csv("Downloads/dayOrthoExpr.csv", row.names=1))
Y <- as.matrix(read.csv("Downloads/nightOrthoExpr.csv", row.names=1))
```

# Main Function: ManiNetCluster

In our package, we provide multiple functions that users can use for separately constructing networks, detecting modules, projecting data into a manifold. But, for convenience, we provide the wrapper function, called ManiNetCluster, which takes two matrices describing the gene expression time series (where rows are genes and columns are timepoints) as inputs and output the projected data in a common manifold and the modules into which the genes are clustered. Users must also provide the corresponding matrix encapsulating the correspondence between two datasets (such as ortholog information). To specify such a corresponding matrix, users must use the class Correspondence provided in our package. The usage is easy as follow: (Here, we use an identity matrix for correspondence since the datasets include the same set of genes in different timepoints)

```
n <- nrow(X) # the number of genes in the dataset
corr <- Correspondence(matrix=diag(n))
```

Other parameters could be tunes are the names of datasets, the dimension of manifold to output, the method, the number of nearest neighbors for approximated graph construction, and the number of modules users want to output. The example use of the function is as follow:

```
df <- ManiNetCluster(X,Y,nameX='light',nameY='dark',corr=corr,d=3L,method='linear manifold',k_NN=6L,k_medoids=60L)
```

Then we could add the locid of genes to the output dataframe by using this line of code:

```
df$id <- c(read.csv("Downloads/dayOrthoExpr.csv")[,1], read.csv("Downloads/nightOrthoExpr.csv")[,1])
```

In the code above, we would like to specify the names of datasets as ‘light’ and ‘dark’, the correspondence matrix is identity as calculated previously; the dimension is 3, the method using is linear manifold alignment; number of nearest neighbors is 6; and numbers of modules is 60. Other methods could be used are: \* ‘cca’: canonical-correlation analysis \* ‘manifold warping’: manifold warping \* ‘nonlinear manifold aln’: nonlinear manifold alignment \* ‘nonlinear manifold warp’: nonlinear manifold warping

The output is the dataframe as follow:

|  | Val1 | Val2 | Val3 | data | module | id |
| --- | --- | --- | --- | --- | --- | --- |
| 1 | -2.6434931 | -0.146657079 | 0.008016877 | light | 15 | Cre01.g002200 |
| 2 | -1.6242314 | 0.302633402 | 0.080689364 | light | 19 | Cre01.g002350 |
| 3 | -1.4356133 | -0.768342141 | 0.001276283 | light | 27 | Cre01.g003376 |
| 4 | -4.0854834 | -0.415335251 | -0.204782870 | light | 13 | Cre01.g007051 |
| 5 | -1.4089036 | 0.462236245 | -0.069898261 | light | 8 | Cre01.g008000 |
| 6 | -1.2170322 | -0.784766106 | -0.205207899 | light | 22 | Cre01.g010848 |
| … | … | … | … | … | … | … |

# Visualization and Comparing the non alignment and alignment results

One method to visualize the alignment result is to use gradient color to capture the local alignment. In this method, same genes from two dataset (or genes which has correspondence to each other) are depicted by the same color. We fix one dataset and color the genes by sorting the expression level of genes by increasing order as in this code:

```
df1 <- df[df$data == 'light',]
df2 <- df[df$data == 'dark',]
df1 <- df1[order(df1$Val1,df1$Val2,df1$Val3), ]
df2 <- df2[match(df1$id, df2$id), ]
```

We use the rgl library for live visualization of data points by points:

```
pal <- rainbow(n)
library(rgl)
for (i in 1:n) {
  with(df1[i,],points3d(Val1,Val2,Val3,size=7,col=pal[i], alpha=.1))
}
```

Using the above, the data points of dataset 1 will form a rainbow cloud as depicted in the figure below:

|  |
| --- |
| *light period gene expression when applying linear manifold* |

If the two datasets are well aligned, we will see the two things: (1) the range of color will keep the same order, and (2) the scale of the plot will approximate the scale of dataset 1 plot. This is because the objective function of manifold alignment/warping tries to minimize both local similarity (depicted by color of the neighborhood) and the global distance between two datasets (depicted by the range of plot). The code and the plot are as follows:

```
for (i in 1:n) {
  with(df2[i,],points3d(Val1,Val2,Val3,size=7,col=pal[i]))
}
```

|  |
| --- |
| *dark period gene expression when applying linear manifold* |

Additionally, we can plot them in the same coordinators as follow:

|  |
| --- |
| *light and dark period gene expression when applying linear manifold* |

From these plots, it is apparent that the alignment is good because the range of color (local similarity) and the scale of plot (global distance) is preserved. We can compare this alignment result with unalignment one. To plot the original datasets we use PCA, the code is as follows:

```
pca_coordinatesX=prcomp(X)$x
df1 <- data.frame(pca_coordinatesX[,1:3])
names(df1) <- c('Val1', 'Val2', 'Val3')
df1$id <- read.csv("Downloads/dayOrthoExpr.csv")[,1]

pca_coordinatesY=prcomp(Y)$x
df2 <- data.frame(pca_coordinatesY[,1:3])
names(df2) <- c('Val1', 'Val2', 'Val3')
df2$id <- read.csv("Downloads/nightOrthoExpr.csv")[,1]

df1 <- df1[order(df1$Val1,df1$Val2,df1$Val3), ]
df2 <- df2[match(df1$id, df2$id), ]

for (i in 1:n) {
  with(df1[i,],points3d(Val1,Val2,Val3,size=7,col=pal[i], alpha=.1))
}

for (i in 1:n) {
  with(df2[i,],points3d(Val1,Val2,Val3,size=7,col=pal[i]))
}
```

Below is the plot of dataset 1:

|  |
| --- |
| *light period gene expression when applying PCA* |

And dataset 2 is as follows:

|  |
| --- |
| *dark period gene expression when applying PCA* |

When plotted simultaneously:

|  |
| --- |
| *light and dark period gene expression when applying PCA* |

This plot demonstrates poor alignment as indicated by absence of scale and color gradient preservation. Thus, the results of alignment, inspected by visualization, is proved to be better than pure PCA.

### Reference

1. J. M. Zones, I. K. Blaby, S. S. Merchant, J. G. Umen, High-Resolution Profiling of a Synchronized Diurnal Transcriptome from Chlamydomonas reinhardtii Reveals Continuous Cell and Metabolic Differentiation. *Plant Cell* **27**, 2743-2769 (2015).
2. Erik L.L.Sonnhammer and Gabriel Östlund. InParanoid 8: orthology analysis between 273 proteomes, mostly eukaryotic Nucleic Acids Res. 43:D234-D239 (2015)
